# PET motion correction using PRESTO with ITK motion estimation

**DOI:** 10.1186/2197-7364-1-S1-A59

**Published:** 2014-07-29

**Authors:** Melissa Botelho, Liliana Caldeira, Juergen Scheins, Nuno Matela, Elena Rota Kops, N Jon Shah

**Affiliations:** Institute of Biophysics and Biomedical Engineering, Science Faculty of University of Lisbon, Kragujevac, Portugal; Institute of Neuroscience and Medicine (INM-4), Forschungszentrum Jülich, Kragujevac, Germany

The Siemens BrainPET scanner is a hybrid MRI/PET system. PET images are prone to motion artefacts which degrade the image quality. Therefore, motion correction is essential. The library PRESTO converts motion-corrected LORs into highly accurate generic projection data [1], providing high-resolution PET images. ITK is an open-source software used for registering multidimensional data [2]. ITK provides motion estimation necessary to PRESTO.

For this study the Utah phantom, a brain phantom and clinical images were used. The data was simulated with different statistics and included scatter and random events. The data was reconstructed using OP-OSEM [3] with 41 iterations. The patient was administrated with [18]F-FDG. The acquisition took about 35min (96min p.i.). Different motions considering translations and rotations with dimensions of 1, 5, 10 and 30mm and degrees in three axes were simulated with PRESTO. In addition to PET images, EPI sequence [3] images were also used. The metric used in ITK registration algorithm was the Mutual Information. Given a fixed image and a moved image, the outputs are six parameters, which represent motion: three translations and three rotations. Then, the images were corrected in PRESTO using these parameters.

**Figure 1:**
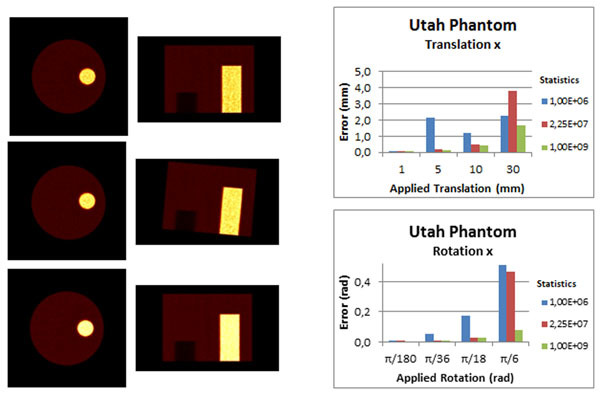
Simulated images in the study with Utah phantom (with a hot and a cold cylinder). On left side of the figure, in the first row, are the acquired PET images of the phantom: first transversal view and then coronal view. The next row shows the simulated images with a translational motion of 5mm (in 3axis) and 5 degree of rotation. The last row corresponds to the corrected images using PRESTO. On the right side of the figure are the graphs representing the absolute errors of ITK values. The graphs represent translation and rotation along the x-axis. The other two axes are not represented here but they have the same behavior as the x-axis.

For low statistics (1E6) and larger motion (30mm/degrees) ITK showed considerable errors (Figure 1). PRESTO using ITK estimated parameters can correct well the moved images. Regarding the PET images, clinical data has bigger errors than phantom data as expected. For MR images ITK errors are smaller than for PET images (Figure 1).

ITK has some limitations, such as, images with low statistics hamper the correction because of the blurring and if the motion is severe the object of study goes out the FOV and then correction is not possible. ITK is a viable image-based correction, which used with PRESTO makes it possible to easily use PET or MR images for motion correction.
